# Childhood adversity and trajectories of health and quality of life in Australian children and adolescents: a latent class analysis

**DOI:** 10.1007/s11136-026-04283-z

**Published:** 2026-06-06

**Authors:** Nirmal Gautam, Mohammad Mafizur Rahman, Rasheda Khanam

**Affiliations:** 1 School of Business, University of Southern Queensland, Toowoomba, QLD 4350 Australia; 2 School of Health and Medical Sciences, University of Southern Queensland, Toowoomba, QLD 4350 Australia; 3 The Centre for Health Research, University of Southern Queensland, Toowoomba, QLD 4350 Australia

**Keywords:** Adverse childhood experiences, Cluster, Health, Quality of life

## Abstract

**Purpose:**

This study aimed to identify adverse childhood experiences (ACEs) clusters and investigate the longitudinal relationships between ACEs clusters and their effects on health and health-related quality of life (HRQoL) in Australian children and adolescents.

**Methods:**

This study used data from the kindergarten cohort of the Longitudinal Study of Australian Children. Latent class analysis was employed to identify ACEs clusters. Generalized estimating equation (GEE) models were used to examine longitudinal associations between ACEs clusters and multiple health outcomes, including general health, mental health, obesity, and HRQoL domains.

**Results:**

The study included 3,089 participants contributing 18,534 observations. Three ACEs clusters were identified: low adversity (65.8%), moderate adversity (25.5%), and high adversity (8.7%). Children in the high-adversity cluster had a higher risk of poorer mental health (IRR = 1.89, 95% CI 1.85–1.92) and suboptimal general health (OR = 1.19, 95% CI 1.17–1.22) compared with those in the low-adversity cluster. Moderate adversity was also associated with elevated risks, although of smaller magnitude. HRQoL scores across social, school, psychosocial, physical, and emotional domains were consistently lower among children exposed to higher adversity.

**Conclusion:**

Distinct ACEs clusters were associated with differences in health and HRQoL among Australian children and adolescents. Identifying ACEs patterns provides valuable insights for developing targeted prevention and intervention strategies aimed at mitigating the long-term health and psychosocial consequences of childhood adversity.

**Supplementary Information:**

The online version contains supplementary material available at 10.1007/s11136-026-04283-z.

## Introduction

Childhood serves as a critical foundation for an individual’s future life [1, 2]. However, this formative stage is alarmingly vulnerable to the detrimental effects of adverse childhood experiences (ACEs). ACEs include forms of child abuse (physical, psychological, and sexual) and dysfunctional household environments characterized by domestic violence, substance abuse, mental health issues, and alcohol and drug misuse. A substantial body of evidence shows that children exposed to ACEs are at increased risk for chronic diseases, psychological disorders [3–8], and diminished quality of life across the life span [9, 10].

The life course health development (LCHD) framework conceptualizes health as a dynamic process shaped by cumulative exposure and sensitive developmental periods. This model offers a valuable lens for understanding how ACEs, especially in clustered patterns, can alter children’s developmental trajectories and affect long-term health and health-related quality of life (HRQoL) [11, 12]. Empirical evidence confirms this pathway: the original ACEs study demonstrated a dose‒response relationship between the number of ACEs and adult disease burden [13], and a meta-analysis revealed substantially elevated risks for multiple health outcomes among individuals exposed to several ACEs [4, 14]. When ACEs are examined as clusters rather than simple counts, the developmental picture sharpens; United Kingdom population data link a “high-adversity” latent class to markedly lower adult QoL scores [15], and recent analyses of adolescents in residential care have revealed that specific abuse-neglect clusters predict poorer psychosocial and physical QoL domains [16]. Together, these findings illustrate how LCHD both explains and is empirically reinforced by ACEs clustering research, providing a coherent developmental framework for integrating timing, cumulative adversity and quality-of-life outcomes in children and adolescents.

While cumulative ACEs scores have been widely used, they oversimplify the diverse and complex nature of childhood adversity [17, 18]. Individuals with the same ACEs count may experience fundamentally different combinations of adversities, which can vary markedly in meaning, timing, and developmental impact. Latent class analysis (LCA), a person-centered statistical method, offers a more nuanced approach by identifying unobserved subgroups with distinct patterns of co-occurring ACEs [17–20]. Unlike cumulative scores, LCA captures heterogeneity in exposure profiles, allowing adversities to cluster in qualitatively distinct ways rather than along a single severity continuum. This classification allows for better stratification of risk and the design of targeted interventions. By distinguishing between different configurations of adversity (e.g., family conflict–dominant vs multi-domain adversity), LCA advances theoretical understanding of how ACEs operate across the life course, and informs evidence-based policymaking [17, 20, 21].

In light of the preceding evidence, this study aims to address the limitations of the previous literature and to provide valuable insights into longitudinal dimensions of ACEs as well as the relationships between ACEs cluster membership and health and health-related quality of life among Australian children and adolescents. Therefore, the first aim of this study was to identify ACEs clusters and investigate the relationships between ACEs cluster membership and health and health-related quality of life among Australian children and adolescents using data from a 14-year follow-up survey of Australian children, the Longitudinal Study of Australian Children (LSAC).

In this study, we focused primarily on the health outcomes: (i) general health, (ii) mental health, (iii) obesity, and HRQoL of children and adolescents. General and mental health describe physical and psychological health status, while HRQoL reflects how children function and feel in their daily physical, emotional, social, and school life [22]. Obesity is considered a physical health outcome influenced by biological stress, family environment, and health behaviors. These outcomes represent different aspects of child health and should not be used interchangeably. Following recent literature [23], we treat them as related but distinct outcomes that may be affected differently by adverse childhood experiences over time.

By identifying and addressing health issues early on, this study contributes to health policy by offering a person-centred classification system that can inform targeted prevention and intervention strategies. Identifying latent ACEs profiles enables policymakers and public health professionals to allocate resources better, design early support systems, and develop trauma-informed practices within schools, healthcare settings, and child protection systems [24].

## Methods and materials

### Study setting, study design, and sample

This study employed data from the kindergarten cohort (K-cohort) of the Longitudinal Study of Australian Children (LSAC), which is a comprehensive and continuing household survey of Australian children initiated in 1999. The LSAC used a multistage cluster sampling method to collect data biennially from children and adolescents. A detailed description of the LSAC methodology can be found elsewhere [25].

The K-cohort initially recruited children aged 4–5 years at Wave 1 and followed the same cohort biennially across seven waves. Accordingly, children were aged 4–5 years at Wave 1, 6–7 years at Wave 2, 8–9 years at Wave 3, 10–11 years at Wave 4, 12–13 years at Wave 5, 14–15 years at Wave 6, and 16–18 years at Wave 7. Thus, the age range “4–7 years” refers to the same cohort of children observed longitudinally across Waves 1 and 2, rather than age variation within a single wave.

During childhood (ages 4–11 years), information on ACEs was obtained primarily through parent or primary caregiver report (P1), who were predominantly biological mothers in approximately 95% of cases. When the parents were unavailable, data were collected from alternate caregivers, such as fathers, grandparents, adoptive parents, and stepparents. For participants aged 12 years and older, the LSAC directly collects data from adolescents using structured questionnaires for both parents and adolescents. Accordingly, ACEs measures in early waves reflect caregiver-reported exposure to household- and family-level adversities, which is appropriate for young children who may lack the cognitive capacity to reliably self-report such experiences. This approach is consistent with established longitudinal cohort methodologies and prior ACEs research using LSAC data.

In this study, ACEs exposures were operationalised using caregiver-reported data collected at Waves 1 and 2 (ages 4–7 years), thereby capturing early-life household and family adversity. To ensure temporal ordering, longitudinal outcomes were examined using subsequent waves. Specifically, general health, mental health (Strengths and Difficulties Questionnaire), obesity (body mass index), and health-related quality of life were assessed repeatedly from Waves 4–7 (ages 10–18 years). Outcomes were treated as repeated measures across adolescence to investigate longitudinal associations between early ACEs clusters and later health trajectories.

Initially, the study included 4,983 participants at Wave 1. The number of participants contributing data at each wave was as follows: Wave 1 (*n* = 4983), Wave 2 (*n* = 4464), Wave 3 (*n* = 4331), Wave 4 (*n* = 4169), Wave 5 (*n* = 3956), Wave 6 (*n* = 3537), and Wave 7 (*n* = 3089). Over the 14-year follow-up period, seven data collection points were analysed (see Appendix C). The analytic sample for latent class analysis included participants with complete data on the selected ACEs indicators, and longitudinal models incorporated all available outcome observations under a missing-at-random assumption. No additional exclusion criteria beyond missing exposure indicators or covariates were applied.

### Dependent variables

This study evaluated various health-related parameters to distinguish between different health status groups among children and adolescents. These parameters included mental health, general health, obesity, and health-related quality of life. These outcome variables were measured from Waves 4–7 (corresponding to participants aged 10–18 years). Mental health (Strengths and Difficulties Questionnaire total score) and HRQoL domain scores were analysed as continuous variables. In contrast, general health was dichotomised as excellent/very good versus good/fair/poor, and body mass index (BMI) was categorised using age- and sex-specific cut-offs to define obesity status. Statistical models were selected according to the measurement scale and distribution of each outcome.

### General health status

Children’s and adolescents’ general health was measured using the following question when the study participants were aged 10–18 years: “In general, how would you rate your (adolescents’) and child’s current health?” The responses were recorded on a 5-point Likert scale (1 = excellent, 2 = very good, 3 = good, 4 = fair, 5 = poor). To facilitate meaningful analysis, we converted the original 5-point Likert scale score into a dichotomous variable. Specifically, responses were coded as 0 = excellent/very good and 1 = good/fair/poor, representing less-than-very-good health (hereafter referred to as suboptimal health).

### Mental health

This study investigated the mental health of children and adolescents aged 10 to 18 years using the Strengths and Difficulties Questionnaire (SDQ), which is available in the LSAC. The SDQ is a well-established tool for assessing mental health [27, 28]. In this study, mental health was assessed using the SDQ [29–31]. The total SDQ score was provided in the LSAC dataset. The total difficulties score (range 0–40) is calculated according to standard SDQ scoring procedures by summing the four problem subscales: emotional symptoms, conduct problems, hyperactivity/inattention, and peer problems [32]. Higher SDQ scores indicate an adverse mental health condition characterized by greater emotional and behavioral difficulties, whereas lower scores indicate better mental health conditions in children and adolescents. The SDQ total score was analysed as a continuous outcome in longitudinal models.

### Obesity

In this study, obesity was assessed using body mass index (BMI), a validated metric calculated by dividing weight in kilograms by height in meters squared. In the LSAC, trained data collectors followed the strict protocols outlined in the user manual. The weight of the participants was measured with Tanita body fat scales, and the height was measured with laser stadiometers between the ages of 10 and 18 years [26]. In addition, the LSAC used the widely recognized age- and sex-specific cut-off points proposed by Cole et al. (2000, 2007) to categorize BMI scores. BMI was categorised into three groups based on predefined cut-offs: normal weight, overweight, and obesity. This three-category variable was analysed as a nominal outcome.

### Health-related quality of life (HRQoL)

The Pediatric Quality of Life (PedsQL) Inventory is a validated questionnaire that measures the HRQoL of children and adolescents [33]. In the LSAC, physical, emotional, school, social, and psychosocial functioning were assessed using the Pediatric Health-related Quality of Life Inventory questionnaire [33, 34]. In this study, HRQoL was assessed at Wave 4 (ages 10–11 years) using parent-reported data and at Waves 5–7 (ages 12–18 years) using adolescent self-reported data, consistent with LSAC procedures. The specific subscales included in our analysis were physical functioning, emotional functioning, school functioning, social functioning, and the psychosocial health summary score. Scores were calculated according to standard PedsQL scoring, ranging from 0 to 100, with higher scores indicating better HRQoL, whereas lower scores indicate poorer functioning. The HRQoL demonstrated strong reliability (alpha ranging from 0.7 to 0.9) and was analysed as a continuous outcome in longitudinal models.

## Independent variables

### Adverse childhood experiences

ACEs are negative events experienced before age 18, including childhood abuse (physical, psychological, or sexual) and household dysfunction, such as parental mental illness, substance use, legal issues, domestic violence, financial hardship, or parental separation [13]. ACEs are well-documented predictors of poor physical and mental health, chronic conditions, and reduced quality of life across the lifespan [4, 13].

In this study, 12 ACEs indicators were derived from the LSAC dataset and were assessed at ages 4–5 (Wave 1) and 6–7 (Wave 2) [35–38]. Each ACEs indicator was recorded as a binary variable (0 = absent, 1 = present) for the analysis.

The ACEs included:

- Physical punishment and neglect: Frequency of punishment and critical comments by parents, dichotomized as “Yes” (any incidence) or “No” (never).
- Hostile parenting: Assessed using items on praise, disapproval, anger during punishment, and difficulty managing the child; coded as “Yes” (any incidence) or “No” (never).
- Intraparental conflict: Disagreements, arguments, or hostility between parents; categorized as “Yes” (sometimes/often/always) or “never”).
- Household dysfunction: Parental or household factors such as heavy drinking, mental illness, legal issues, drug use, bereavement, financial hardship, or parental separation; coded as “1” (present) or “0” (absent). ACEs were measured only during early childhood (ages 4–7 years); adversities occurring later in adolescence were not included in the exposure definition. For more details of all ACEs operationalisation, please Appendix B.

## Potential bias

Cohort studies are generally less susceptible to bias than cross-sectional designs; however, potential selection, information, and confounding biases must still be considered [39]. Fortunately, the LSAC’s rigorous data collection procedures were consistent with international longitudinal standards, which helped minimize non-response and geographic biases [40, 41]. Attrition occurred over follow-up, with sample size declining from Wave 1 to Wave 7. Longitudinal analyses incorporated all available outcome observations under a missing-at-random assumption, and missing values for selected variables were addressed using multiple imputation by chained equations (MICE). Information bias may arise due to differences in informants, as ACEs were caregiver-reported in early childhood (Waves 1–2), whereas several adolescent outcomes were self-reported in later waves (Waves 5–7). As outcome reporting shifted from caregivers in early childhood to self-reports in adolescence, this informant transition was partially accounted for by adjusting for age, gender, and survey wave in longitudinal models. To address confounding, both crude and adjusted models were estimated, with primary models adjusted for age, gender, and survey wave as time-varying covariates. Additional socio-economic and parental characteristics were not included in the primary models due to concerns about potential over-adjustment of variables that may lie on the causal pathway between early ACEs exposure and later health outcomes, which may influence the interpretation of associations. However, residual measurement bias due to differences in informants cannot be fully excluded.

### Statistical analysis

In this study, descriptive statistics were used to summarise the study variables, including frequencies, percentages, and means. ACEs variables were summarised at the participant level to avoid duplication of exposure information across waves, whereas outcome variables were summarised using repeated observations from Waves 4–7 to appropriately reflect their longitudinal measurement structure. Second, LCA was used to identify the latent patterns of ACEs. LCA was selected a priori to capture heterogeneity in ACEs exposure patterns that cannot be adequately represented by cumulative ACEs scores. The LCA included 12 binary ACEs indicators derived from caregiver-reported data at Waves 1 and 2 (ages 4–7 years), coded as “ever exposed” if the adversity was reported at either wave. The analytic sample for the LCA consisted of participants with complete data on all selected ACEs indicators.

Latent class analysis models with two to five classes were estimated using the poLCA package. Model selection was based on the Akaike Information Criterion (AIC), Bayesian Information Criterion (BIC), entropy, class size, and the interpretability of class profiles. The three-class model was selected as the optimal solution based on lower AIC, adequate entropy, and its meaningful and interpretable differentiation of ACE patterns, despite a slightly higher BIC compared with models with more classes. Individuals were assigned to latent classes based on maximum posterior probability, and the resulting ACEs cluster variable was incorporated into subsequent analyses.

Furthermore, generalized estimating equations were used to estimate the longitudinal associations between ACEs cluster membership and health outcomes across waves. Continuous outcomes (mental health and HRQoL domains) were analysed using Gaussian GEE models with an identity link. General health was analysed as a binary outcome (0 = excellent/very good; 1 = good/fair/poor), with excellent/very good serving as the reference category, using a log link within the GEE framework. BMI categories (normal weight, overweight, obesity) were analysed as a three-category nominal outcome, with normal weight specified as the reference category, using multinomial GEE models. An exchangeable working correlation structure was specified for all GEE models to account for within-subject clustering across waves. All models included ACEs classes (with the low-adversity cluster as the reference category) and were adjusted for age, gender, and survey wave as time-varying covariates. Sampling weights and survey design variables (clustering and stratification) were incorporated into the analyses to account for the complex LSAC sampling design. Moreover, missing values for selected variables were addressed via the multiple imputation by chained equations (MICEs) function. All the analyses were performed using the R program (version 4.5.2).

## Results

Latent class models with two to five classes were estimated and compared using model fit indices (Table 1). Although the two-class model showed the lowest BIC, it had low entropy (0.49), indicating poor class separation. The three-class model showed improved fit relative to the two-class solution, as reflected by a lower AIC (7126.36) and higher entropy (0.71), suggesting better class distinction. Although the BIC increased to 7315.39, the three-class model provided a more meaningful representation of heterogeneity in ACE exposure patterns. The four- and five-class models showed only minimal improvements in entropy but were associated with higher AIC and BIC values, indicating reduced parsimony without meaningful improvement in model fit. Detailed model fit information is presented in Table 1.

**Table 1:** Statistical fit metrics for the LCA models

Model	Observation (N)	Likelihood ratio	Goodness of fit	AIC	BIC	Entropy
Class 2	1069	268.76	825.85	7128.77	7253.13	0.49
Class 3	1069	240.35	696.03	7126.36	7315.39	0.71
Class 4	1069	215.54	470.84	7127.55	7381.25	0.72
Class 5	1069	196.13	360.25	7134.13	7452.50	0.75

AIC, Akaike information criterion; BIC, Bayesian information criterion

### Descriptive statistics of the study population

This study included 3,089 participants contributing 18,534 observations across waves (Table 2). The prevalence of adverse childhood experiences varied across domains. Physical punishment (83.2%) and parental neglect (75.9%) were highly prevalent, affecting the majority of participants, while parental legal problems were also frequently reported (85.5%). In contrast, parental separation (7.1%) and parental mental health problems (10.1%) were relatively uncommon. Financial hardship (55.1%) and intra-parental conflict (46.5%) were reported by approximately half of the participants, whereas hostile parenting was less common (34.6%).

**Table 2:** Descriptive statistics of study variables

Variables	Label	Male (*n* = 1556)	Percent (%)	Female (*n* = 1533)	Percent (%)	Total (*n* = 3089)	Percent (%)
Key independent variables							
Punishment	No	228	15.3	267	18.3	495	16.7
	Yes	1266	84.7	1194	81.7	2460	83.2
Hostile parenting	No	901	64.5	905	66.3	1806	65.4
	Yes	496	35.5	460	33.7	956	34.6
Parental neglect	No	352	23.9	350	24.3	702	24.1
	Yes	1118	76.1	1093	75.7	2211	75.9
Intra-parental conflict	No	508	54.8	465	52.1	973	53.5
	Yes	419	45.2	427	47.9	846	46.5
Parental separation	No	1241	92.5	1204	93.3	2445	92.9
	Yes	101	7.5	86	6.7	187	7.1
Financial hardship	No	688	44.4	689	45.4	1377	44.9
	Yes	861	55.6	829	54.6	1690	55.1
Parental legal problem	No	191	15.01	175	14.02	366	14.5
	Yes	1082	84.9	1073	85.9	2155	85.5
Parental alcohol use	No	670	61.5	658	61.4	1328	61.4
	Yes	420	38.5	414	38.6	834	38.6
Parental mental health problem	No	1026	90.3	976	89.5	2002	89.9
	Yes	110	9.7	114	10.5	224	10.1
Death of a family member	No	778	84.1	790	85.9	1568	85
	Yes	147	15.9	130	14.1	277	15
Parental drug use	No	802	86.1	791	86.6	1593	86.4
	Yes	129	13.9	122	13.4	251	13.6
Dependent variables							
General health	Suboptimal health	977	15.9	987	16.3	1964	16.1
	Good health	5171	84.1	5051	83.7	10,222	83.9
Obesity	Normal BMI	130	12	136	13.3	266	12.6
	Overweight	696	64.5	641	62.5	1337	63.5
	Obesity	253	23.4	249	24.3	502	23.8

		Mean (SD)	Mean (SD)	Mean (SD)
Health-related quality of life (HRQoL)	Emotional functioning score	74 (17.6)	74.4 (17.6)	74.2 (17.6)
	Social functioning score	81.5 (18.4)	81.9 (18.1)	81.7 (18.3)
	School functioning score	82.8 (18.3)	82.7 (18.3)	82.8 (18.3)
	Psychosocial health score	78.6 (14.9)	78.9 (14.8)	78.8 (14.9)
	Physical health score	81.3 (17.9)	81.3 (18)	81.3 (17.9)
Strength and Difficulties Questionnaire (SDQ)	Mental health score	7.4 (5.5)	7.5 (5.5)	7.4 (5.5)

Regarding health outcomes, 16.1% of participants were classified as having suboptimal health, with similar proportions observed between males and females. A substantial proportion of participants were classified as overweight (63.5%), followed by obesity (23.8%), with a smaller proportion in the normal BMI category (12.6%). HRQoL scores were generally high across all domains, with minimal differences between males and females. Similarly, the mean mental health score was comparable between males (7.4 ± 5.5) and females (7.5 ± 5.5), indicating no substantial gender differences (Table 2).

### Cluster profiles

Table 3 presents the item-response probabilities of ACEs indicators across the three latent classes. The largest group, Class 1 (labelled as low probability of co-occurrence of ACEs; 65.8%), was characterised by comparatively lower probabilities of severe household adversities, including financial hardship, parental mental health problems, substance use, and family disruption, although high probabilities of certain parenting-related adversities (e.g., physical punishment and hostile parenting) were observed. Class 2 (moderate probability of co-occurrence of ACEs; 25.5%) showed elevated probabilities across multiple domains, including intimate partner violence, financial hardship, parental mental health problems, and household drug use. This class represents a multiple-adversity profile, characterised by co-occurring parenting and household-level adversities.

**Table 3:** Item-response probabilities of ACE indicators across the latent classes

Variables	Category	Clusters for participants (*n* = 3089)		
		Class 1/Low probability of ACEs (*n* = 2034)	Class 2/Moderate probability of ACEs (*n* = 786)	Class 3/High probability of ACEs (*n* = 269)
Physical punishment	No	0	0	1
	Yes	1	1	0
Hostile parenting	No	0.03	0.04	0.06
	Yes	0.97	0.96	0.94
Parental neglect	No	0	0	0.01
	Yes	1	0.99	0.99
Intra-parental conflict	No			
	Yes			
Intimate parental violence	No	0.85	0.47	0.89
	Yes	0.15	0.53	0.11
Financial hardship	No	0.93	0.54	0.89
	Yes	0.06	0.46	0.11
Parental legal problem	No	0.98	0.95	0.95
	Yes	0.02	0.05	0.05
Parental separation	No	0.97	0.86	1
	Yes	0.027	0.14	0
Parental mental health problem	No	0.93	0.82	0.96
	Yes	0.06	0.18	0.04
Parental alcohol use	No	0.63	0.67	0.69
	Yes	0.37	0.33	0.31
Parental drug use	No	0.97	0.74	0.96
	Yes	0.03	0.26	0.04
Death of a household member	No	1	0.86	0.89
	Yes	0	0.14	0.11

ACE indicators were measured as binary (yes/no) variables. Values represent item-response probabilities derived from latent class analysis, reflecting the probability of exposure to each ACE within a given class

Class 3 (high probability of co-occurrence of ACEs; 8.7%) was characterised by consistently high probabilities of parenting-related adversities, including physical punishment, hostile parenting, and parental conflict, but comparatively lower probabilities of broader household adversities such as financial hardship and substance use. Overall, these findings indicate that ACEs cluster into distinct profiles reflecting different combinations of parenting and household adversities, rather than a simple accumulation of exposures.

### Associations between ACEs clusters and health outcomes in children and adolescents

Table 4 shows the associations between ACEs cluster membership from waves 1 to 2 (aged 4–7 years) and health outcomes (general health, mental health, and obesity) from ages 10–18 years (Waves 4–7). ACEs cluster membership was significantly associated with general and mental health, while weak associations were observed for obesity. Children in the moderate-adversity cluster had a higher risk of poor mental health (IRR = 1.17; 95% CI 1.12–1.21) and suboptimal general health (OR = 1.06; 95% CI 1.03–1.09) compared with those in the low-adversity cluster. Similarly, children in the high-adversity cluster showed a substantially increased risk of poorer mental health (IRR = 1.89; 95% CI 1.85–1.92) and suboptimal general health (OR = 1.19; 95% CI 1.17–1.22). ACEs were not strongly associated with obesity outcomes. Moreover, children in the high-adversity cluster had lower odds of being in a higher obesity category (POR = 0.83; 95% CI 0.62–0.99) compared with the low-adversity cluster. No significant association was observed for the moderate-adversity cluster.

**Table 4:** Association between clusters of ACEs (aged 4–7 years) and health outcomes in children and adolescents (aged 10–18 years)

Variables	Mental health	General health	Obesity	
			Overweight	Obesity
	IRR with 95% CI	OR with 95% CI	POR with 95% CI	POR with 95% CI
Age group				
Children (Ref = 1)				
Adolescents	0.90 (0.87–0.90)***	1.09 (1.08–1.11)***	0.09 (0.07–0.11)***	5.37 (4.64–6.20)***
Gender (REF: Male = 1)				
Female	0.88 (0.87–0.90)***	1 (0.99–1.01)	1.29 (1.11–1.48)***	1.03 (0.90–1.18)
Low probability of ACEs (Ref = 1)				
Moderate probability of ACEs: Class 2	1.17 (1.12–1.21)***	1.06 (1.03–1.09)***	0.93 (0.76–1.14)	1.14 (0.95–1.38)
High probability of ACEs: Class 3	1.89 (1.85–1.92)***	1.19 (1.17–1.22)***	0.88 (0.76–1.02)	0.83 (0.69–0.99)*

IRR, Incidence rate ratio; OR, Odds ratio; POR, Proportional odds ratio. Children: aged 3–12 years; adolescents: aged 13–18 years

**Fig. 1:**
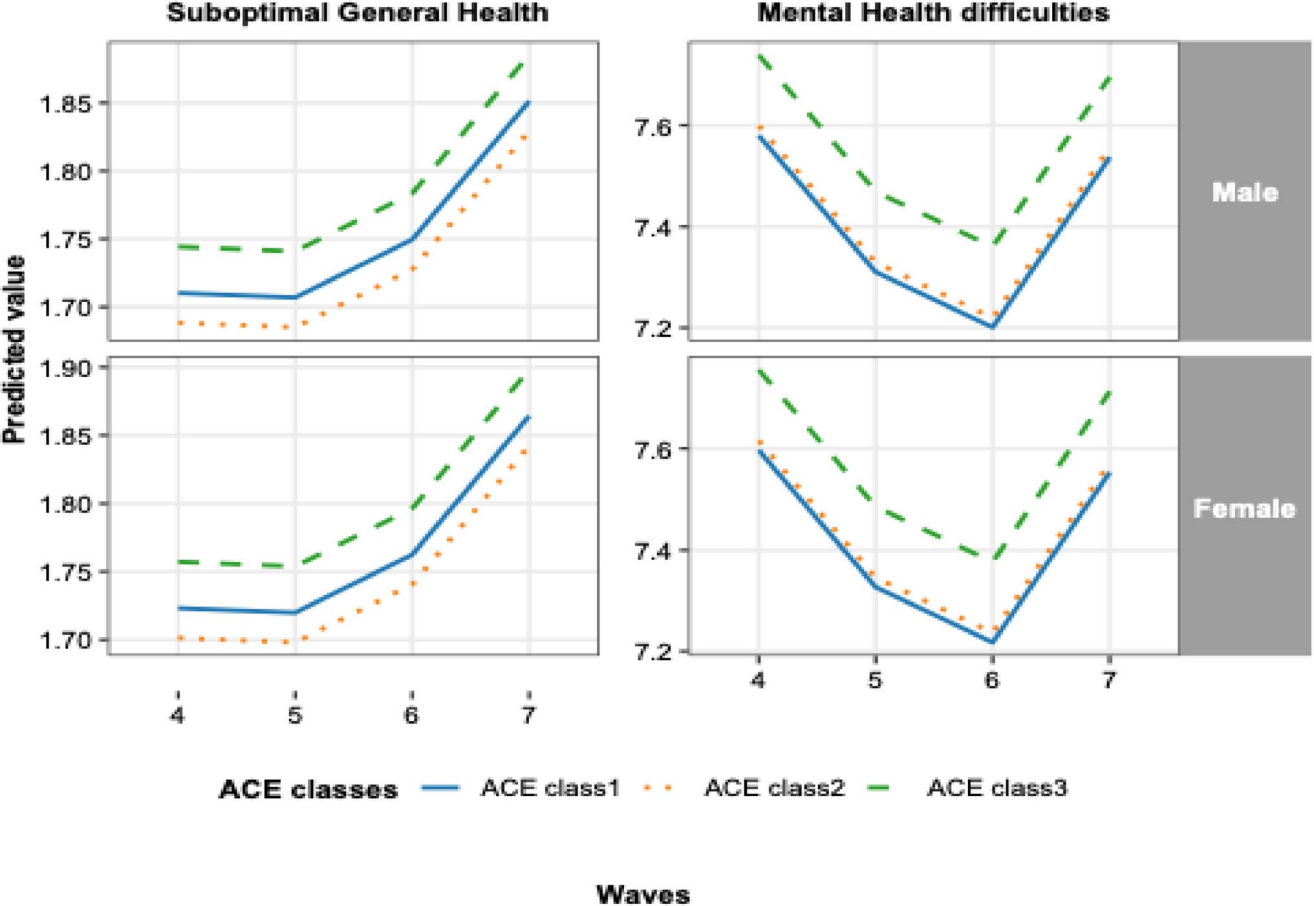
Trajectories of general and mental health across Waves 4–7 by adverse childhood experience (ACEs) classes and gender

Figure 1 shows the trajectories of suboptimal general health and mental health difficulties across Waves 4–7 by ACEs cluster and gender. Overall, children in higher-adversity clusters consistently showed poorer health outcomes than those in lower-adversity clusters. Predicted levels of poor general health increased across waves for both males and females, with the high-adversity cluster showing the highest levels across all waves. Mental health difficulties declined slightly from Wave 4 to Wave 6 before increasing at Wave 7, but children in the high-adversity cluster consistently exhibited higher predicted levels compared with those in the low- and moderate-adversity clusters. The overall patterns were similar for males and females (Fig. 1).

### Associations between ACEs clusters and HRQoL scores in children and adolescents

Higher ACEs cluster membership was associated with lower HRQoL scores across all domains (Table 5). Compared with the low adversity cluster, children in the moderate adversity cluster had lower emotional functioning scores (*β =* − 0.028, 95% CI − 0.038 to − 0.018), as well as lower social functioning (*β =* − 0.011, 95% CI − 0.019 to − 0.003), school functioning (*β =* − 0.010, 95% CI − 0.020 to − 0.001), psychosocial functioning (*β =* − 0.015, 95% CI − 0.023 to − 0.007), and physical functioning score (*β =* − 0.011, 95% CI − 0.019 to − 0.003).

**Table 5:** Association between ACEs cluster (aged 4–7 years) and Health-related quality of life in children and adolescents (aged 10–18 years)

Variables	Health-related quality of life (HRQoL)				
	Emotional functioning Score	Social functioning Score	School functioning Score	Psychosocial health Score	Physical health Score
	Coef (CI)	Coef (CI)	Coef (CI)	Coef (CI)	Coef (CI)
Children: (Ref = 1)					
Adolescents	0.015 (0.011 to 0.019)***	0.002 (− 0.002 to 0.006)	− 0.032 (− 0.036 to − 0.03)***	− 0.000 (− 0.004 to 0.004)	0.018 (0.014 to 0.02)***
Gender (Ref: Male = 1)					
Female	− 0.017 (− 0.021 to − 0.013)***	− 0.003 (− 0.007 to 0.001)	− 0.011 (− 0.015 to − 0.007)***	− 0.008 (− 0.012 to − 0.004)***	− 0.010 (− 0.014 to − 0.006)***
Low probability of ACEs Class1(Ref = 1)					
Moderate probability of ACEs: Class 2	− 0.028 (− 0.038 to − 0.018)***	− 0.011 (− 0.019 to − 0.003)***	− 0.010 (− 0.020 to − 0.001)*	− 0.015 (− 0.023 to − 0.007)***	− 0.011 (− 0.019 to − 0.003)*
High probability of ACEs: Class 3	− 0.089 (− 0.097 to − 0.081)***	− 0.053(− 0.061 to − 0.045)***	− 0.038(− 0.046 to − 0.03)***	− 0.061 (− 0.067 to − 0.055)***	− 0.032 (− 0.038 to − 0.026)***

(1) *P* values are in parentheses: *, ** and *** indicate statistical significance at the 10%, 5%, and 1% levels, respectively, Coef, Coefficient; CI, Confidence interval

Children in the high adversity cluster showed larger reductions across all HRQoL domains, including emotional functioning scores (*β =* − 0.089, 95% CI − 0.097 to − 0.081), social functioning (*β =* − 0.053, 95% CI − 0.061 to − 0.045), school functioning (*β =* − 0.038, 95% CI − 0.046 to − 0.030), psychosocial functioning (*β =* − 0.061, 95% CI − 0.067 to − 0.055), and physical functioning score (*β =* − 0.032, 95% CI − 0.038 to − 0.026). Overall, a clear gradient pattern was observed, with increasing ACEs exposure associated with progressively lower HRQoL scores across all domains.

**Fig. 2:**
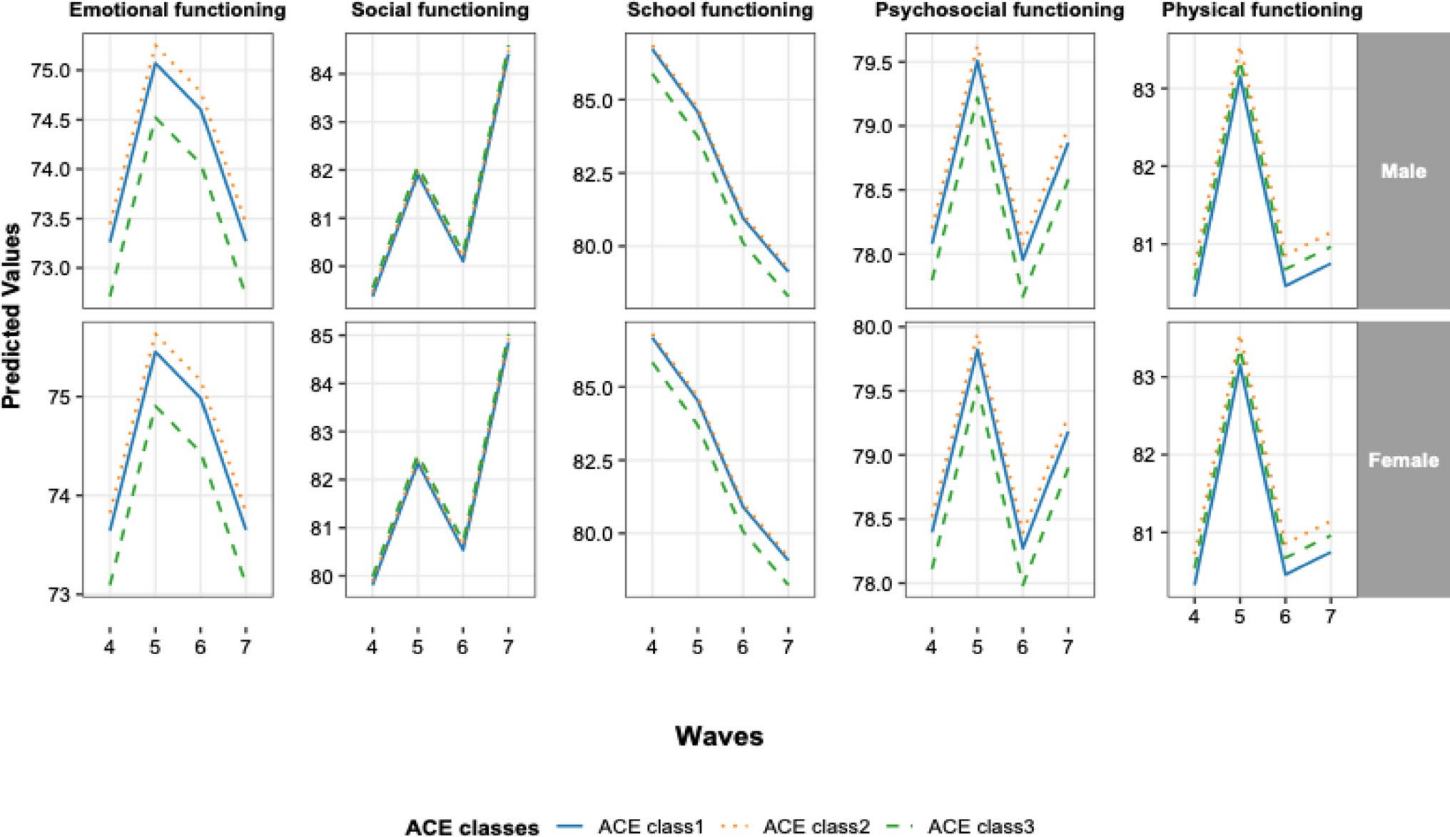
Trajectories of health-related quality of life domains across Waves 4–7 by adverse childhood experience (ACEs) classes and gender

Figure 2 illustrates the trajectories of HRQoL domains across Waves 4–7 by ACEs cluster and gender. Across all domains, children in higher-adversity clusters consistently exhibited lower predicted scores than those in lower-adversity clusters. Emotional and psychosocial functioning increased from Wave 4 to Wave 5, declined at Wave 6, and increased again at Wave 7. Social functioning generally improved across waves, with a slight decrease at Wave 6. School functioning showed a gradual decline over time. Physical functioning increased from Wave 4 to Wave 5, decreased at Wave 6, and partially recovered at Wave 7. These patterns were broadly similar between males and females (Fig. 2).

## Discussion

ACEs were consistently associated with poorer QoL across childhood, adolescence, and adulthood [44, 45]. Guided by life course health development, cumulative risk, toxic stress, and family stress theories, our findings show that distinct ACEs clusters shape persistent health and HRQoL trajectories from childhood through adolescence. Using latent class analysis, we identified heterogeneous patterns of adversity that are differentially associated with general health, mental health, and HRQoL domains. Children in moderate and high-adversity clusters consistently experienced poorer health and lower functioning across waves, supporting the hypothesis that early-life stress disrupts developmental, psychosocial, and biological processes. These findings highlight the importance of person-centred, trauma-informed strategies and early preventive interventions to reduce long-term health inequalities [11, 46].

A key contribution of this study lies in demonstrating the added value of latent class analysis compared with cumulative ACEs scores. While cumulative approaches quantify the overall burden of adversity, they assume that all ACEs exert equivalent effects and fail to distinguish between qualitatively different patterns of exposure. Our findings show that children with similar levels of adversity may belong to distinct latent classes characterized by different combinations of family conflict, maltreatment, and household dysfunction, which are differentially associated with health and HRQoL trajectories. By identifying patterned constellations of ACEs rather than relying solely on exposure counts, LCA provides a more nuanced and developmentally informative understanding of how adversity unfolds across the life course. This person-centered approach aligns with and extends prior ACEs clustering research, which has shown that class-based profiles often explain health outcomes more effectively than cumulative scores alone [15, 16].

Consistent with our hypothesis, exposure to specific ACEs clusters were significantly associated with poorer health outcomes, including general health, mental health, obesity, and diminished HRQoL. More specifically, children in the moderate and high-adversity clusters showed significantly higher risks of poor general health and elevated mental health difficulties compared with those in the low-adversity cluster. The high-adversity cluster demonstrated the strongest associations across outcomes. These findings align with previous evidence showing that clusters involving family conflict, abuse, economic stress, and household mental health problems are linked to poor physical and psychological health and lower QoL [47, 48]. Moreover, greater exposure to adversity was also associated with a progressive decline in HRQoL, supporting earlier findings that higher adversity levels increase the risk of poor health and psychosocial outcomes [49, 50]. These results highlight the need for trauma-informed strategies that address both immediate symptoms and the underlying causes of adversity [51].

In relation to ACEs clusters and general health and mental health among Australian children and adolescents, the findings underscore the biological and psychosocial pathways through which ACEs exert their effects. Chronic exposure to adversity can activate toxic stress responses, disrupting neurological and hormonal systems and impairing brain development [4, 52, 53]. Family conflict and abuse can exacerbate emotional dysregulation and behavioral problems, whereas economic and legal stressors contribute to chronic instability, further increasing the risk of mental and general health problems [54]. Household mental health or substance abuse issues can also perpetuate intergenerational vulnerabilities through both genetic predispositions and learned coping behaviors [55]. These mechanisms help explain the strong associations between high-adversity clusters and poorer mental and physical health observed in this study.

Interestingly, while multiple ACEs clusters were negatively associated with obesity, the patterns were complex. Unlike general and mental health outcomes, the association between ACEs clusters and obesity was not consistently statistically significant. The observed estimates suggested a modest and somewhat inconsistent pattern rather than a clear risk gradient. Some children experiencing moderate adversity appeared more vulnerable to weight gain; however, this difference was not statistically significant. Previous studies suggest that chronic stress related to ACEs may be associated with behavioural factors such as impulse control difficulties and unhealthy dietary patterns, which may increase the risk of obesity across the life course [56–58]. Additionally, chronic stress may influence metabolic processes, including elevated cortisol levels, increased fat storage, and disruptions in insulin regulation [59]. Therefore, the relationship between ACEs and obesity appears to be multifactorial and complex, highlighting the need for cautious interpretation of these findings and the importance of early intervention and support to promote healthier lifestyles.

In the context of ACEs clusters and HRQoL scores in Australian children and adolescents, our findings also revealed that children exposed to any ACEs cluster had significantly lower HRQoL scores across the physical, emotional, social, and school functioning domains. Although both males and females were affected by ACEs exposure, male participants generally reported higher HRQoL scores than female participants across domains; however, these differences should be interpreted cautiously, as they were not formally tested for statistical significance. Exposure to adversity may be associated with impairments in emotional regulation, cognitive function, and physical well-being, while potentially disrupting peer relationships and academic engagement. This finding is consistent with the literature showing that adversity has been associated with neurobiological changes and psychosocial difficulties that compromise development [52, 60, 61]. These disruptions may contribute to sleep problems, nutritional deficiencies, and chronic conditions that persist into adolescence, contributing to persistently lower HRQoL [62–64]. Therefore, exposure to any cluster of adversities was associated with lower quality of life for both males and females, marked by behavioural, emotional, psychological, and social challenges, highlighting the need for targeted support and interventions.

Overall, examining the relationships between ACEs clusters and the health and HRQoL of children and adolescents via the LSAC dataset has significant implications for Australian health and social policy. Identifying distinct ACEs clusters provides an evidence base for more targeted and equitable interventions. Embedding cluster-based screening into health, education, and social service frameworks would allow practitioners, including educators, pediatricians, and social workers, to tailor support to children’s specific profiles of adversity. Such approaches, combined with trauma-informed policies and cross-sector collaboration, can better address the complex needs of vulnerable children.

A key strength of this study is the use of a nationally representative cohort with 14 years of follow-up, enabling robust assessment of longitudinal links between ACEs clusters and health and HRQoL. Advanced statistical methods improved subgroup precision, and validated tools such as the SDQ, standardized HRQoL assessments, and objectively measured BMI increased reliability. However, this study has several limitations: (i) The LSAC dataset lacked data on sexual abuse and parental incarceration, limiting alignment with the original ACEs framework. In addition, the LSAC did not include direct and comprehensive measures of severe physical or emotional neglect. Instead, proxy indicators such as harsh parenting, critical comments, and hostile parenting were used to approximate neglect-related adversity. (ii) Measures of intra-parental conflict and harsh parenting were based on partial scales. (iii) ACEs were assessed only at ages 4–7 years, and later adversities were not captured. (iv) Recall bias may have influenced parent reports. (v) Variability in observations across waves may introduce uncertainty in longitudinal estimates. (vi) Latent class analysis assigns individuals to classes probabilistically, and some degree of classification uncertainty is inherent. (vii) Latent class analysis did not fully account for the complex LSAC sampling design (weights, clustering, and stratification), which may affect the estimation of standard errors and the generalisability of the identified classes. (viii) Attrition across waves may have introduced selection bias if loss to follow-up was not random. Although multiple imputation and longitudinal modelling under a missing-at-random assumption were applied, residual bias may remain. Differences in informants (caregiver-reported ACEs in early childhood and adolescent self-reported outcomes in later waves) may also contribute to measurement variability. (ix) Multiple imputation was performed under the missing-at-random assumption; therefore, results depend on this assumption and on the specified imputation model. Finally, although models were adjusted for age and gender, residual confounding by socio-economic or parental factors cannot be ruled out and may influence the interpretation of the associations between ACEs and health outcomes. Limiting adjustment to avoid over-adjustment may also influence the interpretation of these associations.

## Conclusion

This study demonstrates the utility of integrating LCA with the GEE model to examine longitudinal relationships between distinct ACEs profiles and their associations with health outcomes among Australian children and adolescents. Our findings revealed that multiple clusters of ACEs scores were associated with poor health outcomes, including diminished health-related quality of life, in these populations. Therefore, these findings underscore the urgency of efforts to reduce health inequities and improve the cost-effectiveness of public programs. Health professionals, educators, and child welfare agencies can apply these insights to deliver trauma-informed, targeted support and promote better life-course trajectories.

## Supplementary Information

Below is the link to the electronic supplementary material.

Supplementary Material 1

## Data Availability

The data used are confidential. Interested parties must comply with certain restrictions and sign confidentiality agreements. To request access to the data, individuals should reach out to the Australian Department of Social Services via the following link: https://dataverse.ada.edu.au/dataverse/lsac.

## Abbreviations

ACEs: Adverse childhood experiences
BMI: Body mass index
HRQoL: Health-related quality of life
LSAC: Longitudinal study of Australian children
LCA: Latent class analysis
PedsQoL: Pediatric quality of life
SDQ: Strengths and Difficulties Questionnaire
