# Supplementary material for: Childhood adversity and trajectories of health and quality of life in Australian children and adolescents: a latent class analysis
Source: Qual Life Res. 2026 Jun 6;35(7):171. doi: 10.1007/s11136-026-04283-z (PMC13242438; doi:10.1007/s11136-026-04283-z)
Supplement: Supplementary file 1 — Supplementary Material 1 [file 11136_2026_4283_MOESM1_ESM.docx]

**Appendix A**

**STROBE Statement—Checklist of items that should be included in reports of *cohort studies.***

|  | Item No | Recommendation | Page No |
| --- | --- | --- | --- |
| **Title and abstract** | 1 | Provide in the abstract an informative and balanced summary of what was done and what was found | Page 2: L. No. 17 to 27 |
| Introduction | | | |
| Background/rationale | 2 | Explain the scientific background and rationale for the investigation being reported | Page 3: L. No 42 to 61 |
| Objectives | 3 | State specific objectives, including any prespecified hypotheses | Page 3: L. No. 62 to 66 |
| Methods | | | |
| Study design | 4 | Present key elements of study design early in the paper | Page 3: L. No. 75 to 78 |
| Setting | 5 | Describe the setting, locations, and relevant dates, including periods of recruitment, exposure, follow-up, and data collection | Page 3: L. No. 78 to 87 |
| Participants | 6 | Give the eligibility criteria, and the sources and methods of selection of participants. Describe methods of follow-up | Page 3: L. No. 78 to 81 & 84 to 86 |
| Variables | 7 | Clearly define all outcomes, exposures, predictors, potential confounders, and effect modifiers. Give diagnostic criteria, if applicable | Page 3 to 5: L. No. 89 to 164 |
| Bias | 8 | Describe any efforts to address potential sources of bias | Page 5 L. No166 to 171 |
| Study size | 8 | Explain how the study size was arrived at | See Appendix A |
| Quantitative variables | 9 | Explain how quantitative variables were handled in the analyses. If applicable, describe which groupings were chosen and why | Page 3 to 5:  L. No. 96 to 101,  L. No.103 to 109,  L. No. 111 to 125,  L. No. 127 to 146,  L. No. 149 to 164 |
| Statistical methods | 10 | Describe all statistical methods | Page 5: L. No. 173 to 187 |
| Results | | |  |
| Participants | 11 | Report numbers of individuals at each stage of study—eg numbers potentially eligible, examined for eligibility, confirmed eligible, included in the study, completing follow-up, and analysed | See Appendix A |
| Descriptive data | 12 | Give characteristics of study participants (eg demographic, clinical, social) information | See Table 2 |
| Outcome data | 13 | Report average numbers of outcome events | Page 5: L. No. 197 to 208 |
| Main results | 14 | Give unadjusted estimates and, if applicable, confounder-adjusted estimates and their precision (e.g., 95% confidence interval). | Page 6: L. No 222 to 252 |
| Other analyses | 15 | Report other analyses done— e.g., LCA analyses | Page 6: L. No. 211 to 219 |
| **Discussion** | | | |
| Key results | 16 | Summarise key results with reference to study objectives | Page 6 to 7  L. No. 257 to 259  L. No. 260 to 294 |
| Limitations | 17 | Discuss the limitations of the study | Page 7: L. No. 305 to 309 |
| Generalisability | 18 | Discuss the generalisability (external validity) of the study results | Page 7: L. No. 295 to 301 |
| **Other information** |  |  |  |
| Funding | 19 | Give the source of funding and the role of the funders for the present study and, if applicable, for the original study on which the present article is based | NA |

**Appendix B: Operationalisation of the 12 ACE Indicators**

| ACE Indicator | **Operationalized** | **Original Scale** | **Cut off point** |
| --- | --- | --- | --- |
| Physical punishment | Frequency of punishing the child (Waves 1–2) | 1 = Never/Almost never  2 = Less than half the time  3 = About half the time  4 = More than half the time  5 = All the time | ≤ 2 = 0 (not exposed)  > 2 = 1 (exposed) |
| Hostile parenting | Frequency of disapproval, anger during punishment, or low praise (Waves 1–2) | 1 = Never/Almost never  2 = Less than half the time  3 = About half the time  4 = More than half the time  5 = All the time | ≤ 2 = 0 (not exposed)  > 2 = 1 (exposed) |
| Parental neglect | Telling the child he/she is bad or not as good as others (Waves 1–2) | 1 = Never/Almost never  2 = Less than half the time  3 = About half the time  4 = More than half the time  5 = All the time | ≤ 2 = 0 (not exposed)  > 2 = 1 (exposed) |
| Intra-Parental conflict | Disagreements about child-rearing, stressful conversations, arguments, anger/hostility between partners (Waves 1–2) | 1 = Never/Almost never  2 = Less than half the time  3 = About half the time  4 = More than half the time  5 = All the time | ≤ 2 = 0 (not exposed)  > 2 = 1 (exposed) |
| Financial hardship | Caregiver-reported household financial strain or material deprivation (Waves 1–2) | 1 = Never/Almost never  2 = Less than half the time  3 = About half the time  4 = More than half the time  5 = All the time | ≤ 2 = 0 (not exposed)  > 2 = 1 (exposed) |
| Parental separation | Parental separation or divorce (Waves 1–2) | *0= No*  *1= Yes* | *0= No*  *1= Yes* |
| Parental legal problem | Legal difficulties (arrest, court involvement, incarceration) (Waves 1–2) | *0= No*  *1= Yes* | *0= No*  *1= Yes* |
| Parental alcohol use | Alcohol-related problems within the household (Waves 1–2) | *0= No*  *1= Yes* | *0= No*  *1= Yes* |
| Parental mental health problem | Probable serious mental illness of parent (Waves 1–2) | *0= No*  *1= Yes* | *0= No*  *1= Yes* |
| Death of a family member | Death of parent or close family member (Waves 1–2) | *0= No*  *1= Yes* | *0= No*  *1= Yes* |
| Parental drug use | Illicit drug use or drug-related problems within the household (Waves 1–2) | *0= No*  *1= Yes* | *0= No*  *1= Yes* |

**Appendix C**

**LSAC Participants**

**Wave, Year**

Parents, n= 3537

Children, n = 3537

Parents, n= 3089

Children, n = 3089

Parents, n= 3957

Children, n = 3957

Parents, n= 4169

Children, n = 4169

Parents, n= 4331

Children, n = 4331

Parents, n= 4464

Children, n = 4464

Parents, n= 4983

Children, n = 4983

**Figure 1: Participant diagram**

Wave 1, 2004

Wave 7, 2016

Wave 6, 2014

Wave 5, 2012

Wave 4, 2010

Wave 3, 2008

Wave 2, 2006
